# Menstrual disturbances and its association with sleep disturbances: a systematic review

**DOI:** 10.1186/s12905-023-02629-0

**Published:** 2023-09-01

**Authors:** Bomin Jeon, Jihyun Baek

**Affiliations:** 1 College of Nursing, University of Iowa, Iowa city, USA; 2 College of Nursing, Research Institute of Nursing Science, Jeonbuk National University, 567 Baekje-daero, Deokjin-gu, Jeonju-si, Jeollabuk-do 54896 Republic of Korea

**Keywords:** Sleep, Sleep disorders, Sleep deprivation, Premenstrual syndrome, Dysmenorrhea, Menstruation disturbances

## Abstract

**Background:**

Menstrual disturbances harm women’s health, and general well-being. As growing evidence highlights the relationship between sleep and menstrual disturbances, it is imperative to comprehensively examine the association between sleep and menstrual disturbance considering the multiple dimensions of sleep. This systematic review aims to identify the association between sleep and menstrual disturbances by evaluating using Buysse’s sleep health framework.

**Methods:**

A comprehensive search of the literature was conducted in PubMed, EMBASE, psychINFO, and CINAHL to identify publications describing any types of menstrual disturbances, and their associations with sleep published between January 1, 1988 to June 2, 2022. Quality assessment was conducted using the Joanna Briggs Institute Critical Appraisal Checklist for Analytical Cross-Sectional Studies. The findings were iteratively evaluated menstrual disturbances and their association with sleep using Buysse’s sleep health framework. This framework understands sleep as multidimensional concept and provides a holistic framing of sleep including Satisfaction, Alertness during waking hours, Timing of sleep, Efficiency, and Sleep duration. Menstrual disturbances were grouped into three categories: premenstrual syndrome, dysmenorrhea, and abnormal menstrual cycle/heavy bleeding during periods.

**Results:**

Thirty-five studies were reviewed to examine the association between sleep and menstrual disturbances. Premenstrual syndrome and dysmenorrhea were associated with sleep disturbances in sleep health domains of Satisfaction (e.g., poor sleep quality), Alertness during waking hours (e.g., daytime sleepiness), Efficiency (e.g., difficulty initiating/maintaining sleep), and Duration (e.g., short sleep duration). Abnormal menstrual cycle and heavy bleeding during the period were related to Satisfaction, Efficiency, and Duration. There were no studies which investigated the timing of sleep.

**Conclusions/Implications:**

Sleep disturbances within most dimensions of the sleep health framework negatively impact on menstrual disturbances. Future research should longitudinally examine the effects of sleep disturbances in all dimensions of sleep health with the additional objective sleep measure on menstrual disturbances. This review gives insight in that it can be recommended to provide interventions for improving sleep disturbances in women with menstrual disturbance.

**Supplementary Information:**

The online version contains supplementary material available at 10.1186/s12905-023-02629-0.

## Background

Menstrual disturbances are a significant health concern for women of reproductive age. In the United States, more than 4.5 million women of reproductive age suffer from at least one gynecological disturbance related to menstruation [1]. The common types of menstrual disturbances include premenstrual syndrome (PMS), dysmenorrhea (painful menstrual periods), heavy menstrual bleeding, and irregularities in the menstrual cycle in women of reproductive age [2–4]. PMS is a discomfort in the late-luteal phase (i.e., premenstrual phase) including emotional, behavioral, and physical symptoms [2, 5]. It is estimated that almost half of women of reproductive age (47.8%) considerably suffer from PMS [6] and approximately 20% experience premenstrual dysphoric disorder (PMDD), which is a moderate to severe form of PMS [7, 8]. Dysmenorrhea is reported by 66.6% of women of reproductive age. Heavy menstrual bleeding, which is defined as excessive menstrual blood loss of more than 80 mL per cycle [9, 10] and irregular menstrual cycle (no menstruation or menstrual interval of less than 21 days or more than 35 days) [11] account for up to one-third of gynecologic office visits [12].

Menstrual disturbances are severe enough to disrupt women’s physical, social, and emotional quality of life. Women with PMS and PMDD experience substantial emotional distress, functional impairments, and decreased quality of life [8, 13], which leads to increased risk of suicidality [14]. Dysmenorrhea may increase the risk of other chronic pains and reduce women’s health-related quality of life [15–17]. Heavy menstrual bleeding can indicate other gynecological disease, such as endometriosis [10, 18]. Untreated heavy menstrual bleeding can interfere with the daily life activities and quality of life of women [19]. Menstrual regularity is known as an indicator of women’s reproductive health and menstrual irregularity is reported to increase several medical conditions, such as infertility, diabetes mellitus, and endocrine disorder [5, 10, 20]. Promptly preventing and treating these menstrual disturbances has the potential to increase the overall quality of life in women of reproductive age.

Although the etiology of menstrual disturbances is unclear, focusing on sleep as a preventive measure of menstrual disturbances can be beneficial because disrupted sleep, such as poor sleep quality, difficulty initiating or maintaining sleep, or short sleep duration are apparent in women who suffer from menstrual disturbances [3, 10, 11, 15, 21–23]. Previous studies have found that each sleep characteristic, including sleep quality, efficiency, and duration, is independently associated with menstrual disturbances. However, it is important to note that our sleep is characterized by more than one characteristic, and each of sleep characteristics is simultaneously present in all individuals [24, 25]. Thus, it is needed to understand the role of multidimensional aspects of sleep in menstrual disturbances.

The Buysse’s framework of sleep health is useful for understanding sleep as a multidimensional concept [24]. This concept provides a holistic framework for sleep; Satisfaction, Alertness during waking hours, Timing of sleep, Efficiency, and Sleep Duration. The framework can provide a comprehensive understanding of the association between sleep and specific health outcomes, such as obesity, substance use, mental health, cardiovascular disease, and neurodegenerative disease [24, 26, 27]. Thus, this systematic review aims to (a) identify the association between sleep and menstrual disturbances by evaluating using the Buysse’s sleep health framework, and (b) describe gaps in the literature on the relationship between these phenomena in the reviewed studies. This review highlights the role of sleep as a modifiable factor in menstrual disturbances and provides basic knowledge of whether assessing and treating sleep is effective in reducing menstrual disturbances in women of reproductive age.

## Methods

**Fig. 1:**
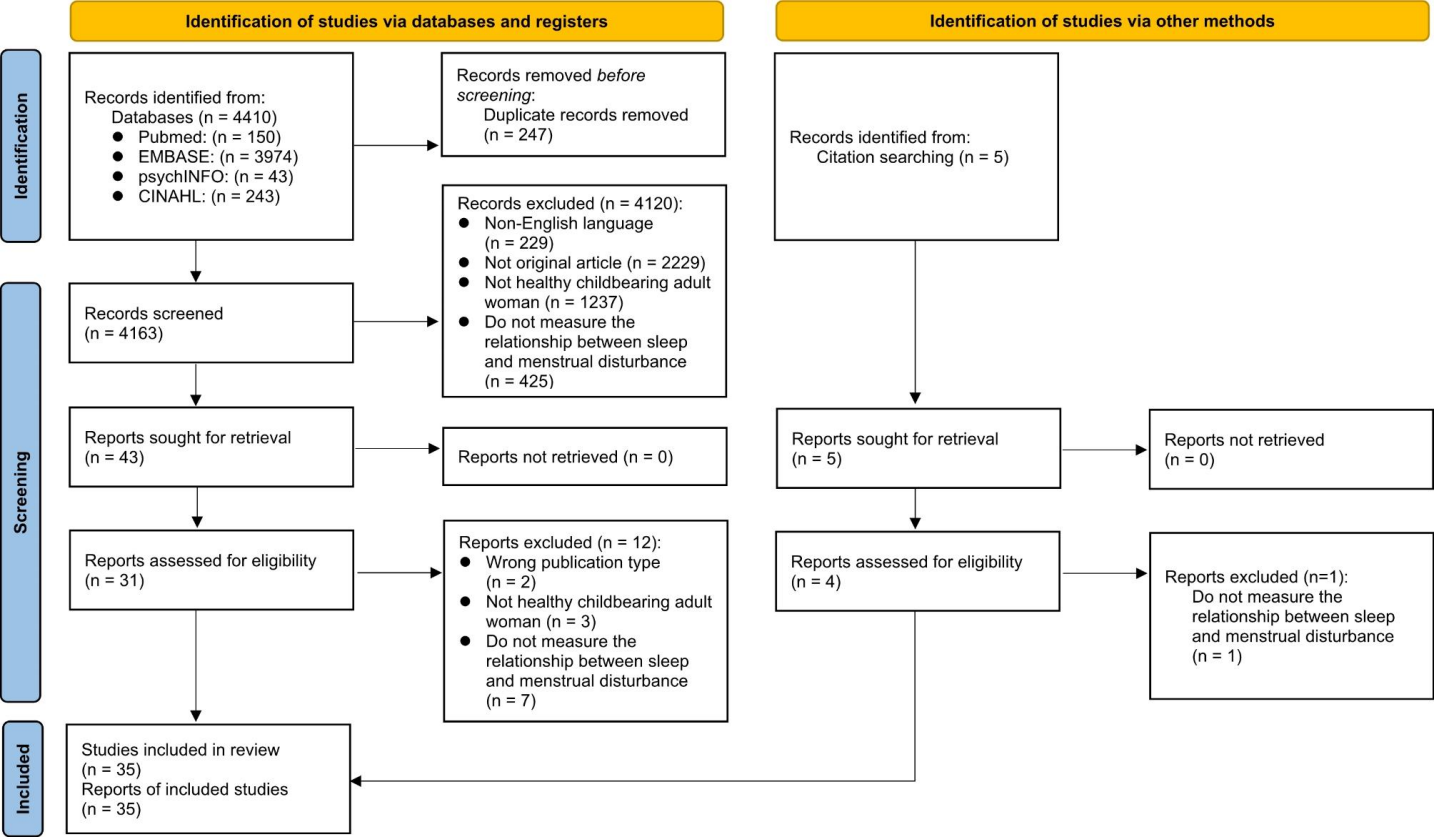
PRISMA 2020 flow diagram for new systematic reviews which included searches of databases, registers and other sources *Consider, if feasible to do so, reporting the number of records identified from each database or register searched (rather than the total number across all databases/registers) **If automation tools were used, indicate how many records were excluded by a human and how many were excluded by automation tools *From*: Page MJ, McKenzie JE, Bossuyt PM, Boutron I, Hoffmann TC, Mulrow CD, et al. The PRISMA 2020 statement: an updated guideline for reporting systematic reviews. BMJ 2021;372:n71. doi: 10.1136/bmj.n71. For more information, visit: http://www.prisma-statement.org/

Based on the purpose of the study, inclusion and exclusion criteria are described below and the entire study selection process and the rationales for exclusion are shown in Fig. 1. This systematic review follows the protocol of Preferred Reporting Items for Systematic Reviews and Meta-Analysis (PRISMA) guidelines [28]. This study was registered in the International Prospective Register of Systematic Reviews (PROSPERO; CRD42022308008).

### Search strategies

Studies were selected from searches in four biomedical electronic databases: PubMed, EMBASE, psychINFO and CINAHL. Articles published from January 1, 1988 to June 2, 2022, were included, as 1988 was the first year that the association between menstrual disturbances and sleep appeared in literature. The following key words and combinations were used: (“dyssomnias” OR “sleep” OR “sleep disorder” OR “sleep disturbance” OR “sleep wake disorder”) AND (“menstruation disturbances” OR “menstruation disturbance” OR “menstrual disorders” OR “irregular menstruation” OR “irregular menstruation” OR “menstrual irregularity” OR “menstrual dysfunction”). The search strategy was developed in consultation with a health science librarian. The complete search strategy is provided in Additional file 1. In addition, in order to identify relevant articles missed by the previous search, the citation lists of selected articles were reviewed.

### Eligibility criteria

Studies were included if they (1) were written in English; (2) published in a peer-reviewed journal; (3) data-based articles; (4) included healthy childbearing adult women (greater than 18 years old) as the study population; and (5) examined any type of menstrual disturbances, and their associations with sleep. Studies were excluded if they (1) focused on adolescent female and menopausal women; and (2) were not original articles (e.g., review paper, commentary, gray articles including dissertation, conference paper, abstract or editorials).

### Data extraction and synthesis

Initial screening was independently conducted by the two authors (JB and BJ) through reading titles and abstracts based on the eligibility criteria. Potentially relevant articles retrieved from initial screening were proceeded to full text review. The two authors (JB and BJ) read the full texts of potentially relevant articles and discussed any concerns or discrepancies in the inclusion of articles. After reading articles in full text, the selected articles were analyzed by two independent authors (JB and BJ). One researcher conducted the data extraction (JB) and the extracted data was checked by the other researcher (BJ). Data from the selected articles was organized under the following headings: first author; year; location; sample details (sample size, age); research design; measures for sleep; measures for menstrual disturbances; and study findings. Extracted data were presented in tabular format and arranged sequentially by alphabet (Table 1). The findings were iteratively evaluated menstrual disturbances and their association with sleep using Buysse’s sleep health framework [24]. Articles were clustered based on the type of menstrual disturbances: (1) PMS and sleep health; (2) dysmenorrhea and sleep health; and (3) Abnormal menstrual cycle/bleeding and sleep health (Table 2).

**Table 1:** Overview of study characteristics

Author(s)	Study participants	Sample size	Age (range/mean ± SD)	Research design	Measurement/instruments (Sleep)	Measurement/instruments (Menstrual disturbances)			Results	Quality Assessment
						PMS (including premenstrual dysphoria, premenstrual related negative affect symptoms, PMDD)	Dysmenorrhea	Menstrual cycle/bleeding during period		
Abdel-Salam et al. [15]	Female university students	321	18–28y	Cross-sectional	Simple question asking whether having sleep disturbances as associated symptoms of dysmenorrhea	n/a	Simple question asking whether having lower abdominal pain connected with menstrual periods	n/a	43% of women with dysmenorrhea, experienced sleep disturbance as one of dysmenorrhea-related symptoms.	Low
Baker et al. [37]	∙ Women with primary dysmenorrhea ∙ Normal controls	∙ Total: 18 ∙ Primary dysmenorrhea: 10 ∙ Normal controls: 8	∙ Primary dysmenorrhea: 23.0 ± 5.0 ∙ Normal controls: 20.0 ± 1.0	Cross-sectional	∙ Sleep architecture (polysomnography) ∙ 100 mm VAS for sleep quality with anchor points of “worst possible” and “best ever”	n/a	Having painful uterine cramps, near and during menstruation without any menstrual-associated mood disturbances	n/a	∙ Women with primary dysmenorrhea had worse sleep quality than normal controls. ∙ Women with primary dysmenorrhea had decreased sleep efficiency than normal controls.	Moderate
Baker et al. [39]	∙ Women with severe PMS ∙ Normal controls	∙ Total: 21 ∙ Severe PMS: 9 ∙ Normal controls: 12	∙ Severe PMS: 28.0 ± 6.0 ∙ Controls: 31.0 ± 5.0	Cross-sectional	∙ Sleep architecture (polysomnography) ∙ PSD (subjective sleep quality)	∙ Penn DSR Form ≥ 80 or at least 50% of an increase in Penn DSR scores during premenstrual phase (Severe PMS)	n/a	n/a	∙ Women with severe PMS reported worse sleep quality than normal controls. ∙ Women with severe PMS had a decreased delta incidence, and increased theta incidence and amplitude in sleep architecture compared with normal controls.	High
Baker et al. [42]	∙ Women with severe PMS ∙ Women with minimal symptoms	∙ Total: 36 ∙ Severe PMS: 18 ∙ Minimal symptoms: 18	∙ Severe PMS: 30.5 ± 7.6 ∙ Minimal symptoms: 29.2 ± 7.3	Cross-sectional	∙ Sleep architecture (polysomnography) ∙ Modified version of the PSD (subjective sleep quality) ∙ 100 mm VAS for sleep quality with anchor points of “very bad” and “very good” ∙ 100 mm VAS for restless of sleep with anchor points of “very restless” and “not at all restless” ∙ 100 mm VAS for refreshing feeling on awakening with anchor points of “not at all refreshed” and “very refreshed” ∙ 100 mm VAS for morning alertness with anchor points of “not at all alert” and “extremely alert.”	∙ Penn DSR Form ≥ 80 or at least 50% of an increase in DSR scores during premenstrual phase (Severe PMS) ∙ Met the diagnostic criteria (DSM-IV) for PMDD	n/a	n/a	Women with severe PMS had more SWS and slow wave activity than those with minimal symptoms.	High
Çaltekin et al. [31]	∙ Women with primary dysmenorrhea ∙ Normal controls	∙ Total: 102 ∙ Primary dysmenorrhea: 55 ∙ Normal controls: 47	∙ Primary dysmenorrhea: 23.0 ± 5.0 ∙ Normal controls: 20.0 ± 1.0	Cross-sectional	∙ ESS (daytime sleepiness) ∙ ISI (insomnia severity) ∙ PSQI (subjective sleep quality) ∙ Berlin Questionnaire (obstructive sleep apnea) ∙ International Restless Legs Syndrome Study Group diagnostic criteria (restless leg syndrome)	n/a	∙ Having history of painful menstruation ∙ Severity of dysmenorrhea: VAS	n/a	∙ Women with primary dysmenorrhea experienced worse sleep quality, greater daytime sleepiness, and greater insomnia symptoms than normal controls. ∙ Among women with primary dysmenorrhea, poor sleep quality and daytime sleepiness were risk factors of insomnia. ∙ Severity of pain was significantly correlated with sleep quality, insomnia severity in women with primary dysmenorrhea.	High
Cheng et al. [43]	∙ PMS patients ∙ Normal controls	1,699	∙Total: 21.58 ± 4.00 ∙PMS patients: 21.67 ± 3.80 ∙Normal controls: 21.51 ± 4.13	Cross-sectional	PSQI ≥ 6 (subjective poor sleep quality)	∙ PMS: at least one of symptoms in the following instrument are noted 1-week before menstruation and subside within a few days after onset of menstruation and disappear after menstruation: ∙ Premenstrual Symptom Questionnaire	n/a	n/a	∙ Poor sleep quality was found in the 60.5% of the PMS patients with PMS, and 40.7% of normal controls. ∙ Poor sleep quality significantly increased the risk of PMS (OR = 1.89; 95% CI [1.51–2.36]).	High
Chuong et al. [36]	∙ PMS patients ∙ Normal controls	∙ Total: 9 ∙ PMS patients: 3 ∙ Normal controls: 6	∙ PMS patients: 32.0 ± 1.2 ∙ Normal controls: 30.5 ± 1.6	Cross-sectional	Sleep architecture (polysomnography)	Symptomatic: at least 30% of an increase in scores for one of the mood-related symptoms and one of the somatic symptoms of PMTS during premenstrual phase	n/a	n/a	There were no significant differences in sleep architecture between PMS patients and normal controls.	High
Conzatti et al. [53]	∙ PMS patients ∙ Normal controls	∙ Total: 121 ∙ PMS: 69 ∙ Normal controls: 52	24–38y	Cross-sectional	∙ ESS > 10 (excessive daytime sleepiness) ∙ PSQI > 5 (subjective poor sleep quality)	∙ PSST for the screening of PMS ∙ DRSP to confirm PMS diagnosis	n/a	n/a	∙ The risk of poor sleep quality was two times higher in women with PMS than normal controls (OR = 3.057; 95% CI 1.44–6.45). ∙ Women with PMS had greater scores in ESS than normal controls.	High
Erbil and Yucesoy [51]	Female university students	313	20.5 ± 1.7	Cross-sectional	PSQI > 5 (subjective poor sleep quality)	PMSS	n/a	n/a	∙ Poor sleep quality was associated with multiple symptoms of PMS including depressive feelings, irritability, appetite changes, and sleep changes ∙ Among multiple PMS symptoms, sleep changes were the strongest predictor of poor sleep quality, followed by depressive thoughts, depressive mood, bloating. ∙ Greater PMSS scores was associated with poor sleep quality.	High
Jang et al. [55]	Female undergraduate nursing school students	304	19 − 21y 20.6 ± 1.7	Cross-sectional	∙ Sleep duration ∙ PSQI (subjective poor sleep quality)	S-PAF	n/a	n/a	∙ Sleep quality was positively correlated with PMS symptoms. ∙ The correlation between sleep duration and PMS symptoms was not significant.	High
Kamel et al. [32]	Women in the reproductive age	688	25.6 ± 7.7	Cross-sectional	PSQI > 5 (subjective poor sleep quality)	PSS ≥ 80 points or above	n/a	n/a	∙ Poor sleep quality increased the severity of PMS. ∙ 92.3% of women with PMS experienced poor sleep quality, whereas only 7.6% of woman without PMS experienced poor sleep.	High
Kang et al. [11]	Newly employed female nurses working shifts who had the menstrual cycle regularity at baseline	287	Approximately 24	Prospective longitudinal	ISI > 7 (having insomnia)	n/a	n/a	Simple question for menstrual cycle (Yes/No): “Has your menstruation cycle period ever been shorter than 21 days or longer than 35 days, at least once during the last 6 months?” (Menstrual cycle < 21 days or > 35 days: menstrual cycle irregularity)	∙ The incidence of menstrual cycle irregularity in women with insomnia at baseline was 2-times greater than those without insomnia at baseline (OR = 2.05, 95% CI [1.12–3.77]). ∙ The prevalence of menstrual cycle irregularity in women with insomnia at baseline was 3-times greater than those without insomnia at baseline (OR = 3.05, 95% CI [1.81 − 5.13]).	High
Kennedy et al. [10]	Women who had experienced menstrual cycle within the last 12 months	579	22–60y 32.4 ± 5.6	Cross-sectional	∙ ESS (daytime sleepiness) ∙ ISI (insomnia severity) ∙ PSQI (subjective sleep quality) ∙ Sleep duration (≤ 6 h; 6–8 h; ≥ 9 h)	n/a	n/a	∙ Simple question for menstrual cycle: “How regular is your period?” (very regular; mostly regular; fairly regular; not regular) ∙ Simple question for amount of bleeding during menstruation: “How much bleeding do you usually experience during your period?” (very heavy; heavy; medium; light; very light)	∙ Compared to the students who slept between 6 to 8 h, ≤ 6 h was associated with heavier bleeding (OR 1.46, p = 0.026), and greater cycle irregularity (OR 1.44, p = 0.031). ∙ Worse sleep quality was associated with greater cycle irregularity (OR 1.05, p = 0.022). ∙ Long sleep duration insomnia severity, and daytime sleepiness were not associated with menstrual regularity and amount of bleeding during menstruation.	Moderate
Khazaie et al. [48]	∙ PMDD patients ∙ Normal controls	262	∙ PMDD: 23.4 ± 4.1 ∙ Normal controls: 23.7 ± 4.7	Cross-sectional	PSQI > 5 (subjective poor sleep quality)	Affirmative response on PSST questions for PMDD following the diagnostic criteria (DSM-IV-TR)	n/a	n/a	Women with PMDD experienced greater level of poor sleep quality than normal controls.	High
Kim et al. [49]	∙ Women with menstrual cycle irregularity ∙ Women without menstrual cycle irregularity	4,445	∙ Menstrual cycle irregularity: 32.3 ± 0.5 ∙ Without menstrual cycle irregularity: 34.9 ± 0.2	Cross-sectional	Simple question for sleep duration: “How many hours do you sleep on average?” (≤ 5 h; 6–7 h; ≥ 8 h)	n/a	n/a	∙ Simple question for menstrual cycle (Yes/No): “Is your menstrual cycle currently regular?“ ∙ Severe menstrual irregularity indicates the interval between menstruations of greater than 3 months	Short sleep duration (≤ 5 h) increases the risk of having severe menstrual cycle irregularity as two times higher than regular sleep duration (6–8 h; OR = 2.67, 95%CI [1.35–5.27]).	Moderate
Lee et al. [35]	Healthy and presumably ovulating women	13	25–35y	Cross-sectional	Sleep architecture (polysomnography)	Symptomatic: at least 30% of an increase in score of following instruments during premenstrual phase: ∙ Profile of Mood States ∙ Modified Woods Women’s Health Diary	n/a	n/a	Women with premenstrual related negative affect symptoms reported significantly less delta sleep than those without premenstrual related negative affect symptoms.	Moderate
Lim et al. [47]	Female healthcare workers, healthy referral patients	231	21–45y 31.6 ± 5.6	Cross-sectional	Sleep duration (< 6 h; ≥ 6 h)	n/a	n/a	∙ Short menstrual cycle: <25 days ∙ Normal menstrual cycle: 25–34 days ∙ Long menstrual cycle: 35 days	Women reporting fewer than 6 h of sleep were more likely to report abnormal (short or long) menstrual cycle lengths (OR = 2.1; 95% CI [1.1 to 4.2]).	Moderate
Lin et al. [52]	∙ PMDD patients ∙ Normal controls	∙ Total: 196 ∙ PMDD: 100 ∙ Normal controls: 96	∙ PMDD: 24.8 ± 3.3 ∙ Normal controls: 24.8 ± 3.4	Cross-sectional	PIRS > 20 (having insomnia)	PMDD: Affirmative response on PSST questions for PMDD following the diagnostic criteria (DSM-IV-TR) + at least 30% of an increase in scores for PMDDSQ during premenstrual phase	n/a	n/a	∙ Women with PMDD had a higher incidence of insomnia than normal controls. ∙ The exacerbation of insomnia in women with PMDD was greater in the premenstrual phases than in control groups ∙ PMDD severity was positively associated with insomnia severity in women with PMDD.	High
Maher et al. [54]	Women in the reproductive age	1,335	29–38y 34	Cross-sectional	PSQI ≥ 5 (subjective poor sleep quality)	n/a	n/a	Unknown	∙ Sleep quality was associated with the change of overall menstrual cycle (OR 1.11, 95% CI 1.048–1.178), and the increase of missed periods during pandemic (OR 1.11, 95% CI 1.029–1.189). ∙ Worse sleep quality was associated with the number of painful periods during pandemic (OR 1.07, 95% CI 1.005–1.130).	Moderate
Matsumoto et al. [56]	Female college student	22	20.5 ± 1.1	Cross-sectional	Sleep duration (< 6 h; ≥ 6 h	Moos MDQ	n/a	n/a	< 6 h of sleep at night is associated with worse PMS symptoms.	High
Mauri et al. [34]	∙ PMS patients ∙ Controls with a significant premenstrual disturbance ∙ Controls without premenstrual disturbance	∙ Total: 40 ∙ PMS patients: 14 ∙ Controls with disturbance: 15 ∙ Controls without disturbance: 11	∙ PMS patients: 35.4 ± 6.5 ∙ Controls with disturbance: 34.2 ± 4.8 ∙ Controls without disturbance: 32.2 ± 8.2	Cross-sectional	PSI (subjective sleep quality, difficulty initiating and/or maintaining sleep, early morning awakenings)	∙ PMS: Seeking medical help d/t severe PMS from PMS clinic ∙ Premenstrual Tension Syndrome Self Rating Scale ≥ 14 (Significant premenstrual disturbance)	n/a	n/a	∙ PMS patients experienced more frequent awakening during sleep and took more time to go back to sleep after an awakening than control groups with/without disturbance. ∙ PMS patients failed to wake up at the expected time and more tired than control groups with/without disturbance in the morning.	High
Mishra et al. [46]	∙ PMDD patients ∙ Normal controls	179	∙ Total: 22.9 ± 2.9 ∙ PMDD: 24.49 ± 2.34 ∙ Normal controls: 21.98 ± 2.85	Cross-sectional	Sleep duration (total number of hours of sleep obtained per 24 h)	Affirmative response on SSQ questions for PMDD following the diagnostic criteria (DMV-IV-TR)	n/a	n/a	∙ Women who had PMDD had significantly shorter sleep duration.	Moderate
Molugulu et al. [59]	Female college student	110	≥ 18y	Cross-sectional	Simple question asking whether having sleep problems	∙ PMS: at least one of somatic/affective/psychological symptoms in the following instrument during premenstrual phase with regular monthly menses: ∙ PMDD: Psychological symptoms following instrument rated as severe and others rated as moderate to severe in addition with at least one somatic symptom rated as severe to moderate: ∙ Pre-Menstrual Severity Screening Tool	n/a	n/a	∙ 57% of women who diagnosed with PMS had sleeping problems. ∙ PMS severity was associated with sleep problems.	Low
Momma et al. [22]	Women with no history of a previous pregnancy and/or childbirth	∙ Athletes: 605 ∙ Non-athletes: 295	19–20y ∙ Athletes: 20.0 ∙ Non-athletes: 20.0	Cross-sectional	Sleep duration	n/a	Dysmenorrhea severity: “none = 0 to heavy pain = 10” (none/mild = 0 to 3; moderate = 4 to 6; severe = 7 to 10)	n/a	Sleep duration was not associated with dysmenorrhea severity in both athletes and non-athletes.	Moderate
Nicolau et al. [50]	∙ PMS patients ∙ Normal controls	230	∙ PMS: 36.1 ± 0.6 ∙ Normal controls: 37.6 ± 1.0	Cross-sectional	∙ PSQI (subjective sleep quality) ∙ ESS (daytime sleepiness) ∙ ISI (insomnia severity) ∙ General sleep questionnaire ∙ Sleep architecture (polysomnography)	Simple question for PMS (Yes/No): “Do you have PMS?”	n/a	n/a	∙ PMS patients experienced poorer sleep quality, a higher perception of unrefreshing sleep, a higher total sleep time, and having threshold insomnia than normal controls ∙ PMS patients has longer sleep duration than those without PMS. ∙ Longer sleep duration and having subthreshold insomnia were associated with PMS. ∙ Daytime sleepiness, sleep latency, time after sleep onset, and sleep efficiency were not associated with PMS.	High
Ozisik Karaman et al. [58]	Medical academy female students	178	No information	Cross-sectional	PSQI > 5 (subjective poor sleep quality)	PSS ≥ 102 points or above	n/a	n/a	Poor sleep quality was found in the 75.6% of the women with PMS, and 58.8% of the women without PMS (*p* < 0.05).	Low
Prabhavathi et al. [13]	Female nursing school students	100	No information	Cross-sectional	∙ PSQI (subjective sleep quality) ∙ ISI (insomnia severity)	Modified Moos MDQ (Mild vs. Moderate)	n/a	n/a	∙ Women with moderate severity of PMS experienced worse sleep quality than those with mild severity of PMS	Moderate
Prabhavathi et al. [57]	Female nursing school students	60	18–20y	Cross-sectional	∙ PSQI (subjective sleep quality) ∙ ISI (insomnia severity)	Modified Moos MDQ (No vs. Mild vs. Moderate)	n/a	n/a	Women with moderate severity of PMS experienced worse sleep quality and had greater insomnia severity than those with mild severity of PMS or without PMS	Moderate
Shao et al. [40]	Female Shift-work nurses	435	29.50 ± 5.43	Cross-sectional	PSQI (subjective sleep quality)	Having premenstrual dysphoria (Yes/No)	Having pain during period (Yes/No)	n/a	∙ Women with premenstrual dysphoria had worse sleep quality than those without. ∙ Women with pain during period had worse sleep quality than those without.	Moderate
Shechter et al. [41]	∙ PMDD patients who indicated insomnia during premenstrual phase ∙ Normal controls	∙ Total: 12 ∙ PMDD patients: 7 ∙ Normal controls: 5	∙ PMDD patients: 32.0 ± 5.72 ∙ Normal controls: 30.4 ± 8.20	Cross-sectional	Sleep architecture (polysomnography)	∙ PMDD: at least 200% of an increase in score of one symptom or at least 100% of an increase in scores of two or more symptoms during premenstrual phase in following instruments: ∙ 100-mm VAS for depressed mood, tension, affective liability, irritability	n/a	Simple question for menstrual cycle length	∙ SWS was significantly increased in PMDD patients than normal controls. ∙ Menstrual cycle length did not differ between PMDD patients and normal controls.	High
Strine et al. [38]	Noninstitutionalized U.S women	11,648	18–55y	Cross-sectional	∙ Simple question for insomnia (Yes/No): “Have you regularly had insomnia or trouble sleeping?” ∙ Simple question for daytime sleepiness (Yes/No): “Have you had excessive sleepiness during the day?”	Simple question for premenstrual syndrome/bothersome cramping/heavy bleeding (Yes/No): “During the past 12 months, have you had any menstrual problems, such as heavy bleeding, bothersome cramping, or premenstrual syndrome (also called PMS)?”			Women with menstrual-related problems were significantly more likely to report frequent insomnia, excessive sleepiness than those without menstrual-related problems over the past 12 months.	Moderate
Unver et al. [33]	Female university students	353	18–31y 21.1 ± 1.8	Cross-sectional	∙ Questionnaire asking sleep latency, frequency of awakening after falling asleep, sleep duration, sleep quality, feeling rested in the morning of the last three days. ∙ ISI ≥ 10 (having insomnia)	n/a	Pain in the waist, inguinal, or abdominal regions which starts in the first six to 12 h of menstruation and lasts for eight to 72 h	n/a	∙ Women with dysmenorrhea experienced greater insomnia symptoms, shorter sleep duration, worse sleep quality and greater feeling of unrested in the morning than those without. ∙ Women with severe dysmenorrhea experienced higher frequency of awakening after falling asleep, worse sleep quality and greater feeling of unrested in the morning than those mild or moderate dysmenorrhea.	Moderate
Woosley and Lichstein [44]	Undergraduate female students	89	18–24y 18.63 ± 0.93	Cross-sectional	∙ PSQI > 5 (subjective poor sleep quality) ∙ ISI (insomnia severity, ≥ 7 indicates having clinical insomnia) ∙ Diagnostic criteria of insomnia following ICSD-II ∙ CSD (sleep latency, wake time after sleep onset, number of awakenings, total sleep time, and sleep efficiency)	n/a	Brief Pain Inventory	n/a	∙ Women with insomnia had worse dysmenorrhea and experienced more interference with daily activities due to dysmenorrhea than those without insomnia. ∙ Insomnia severity was directly associated with dysmenorrhea severity ∙ Insomnia severity and sleep quality was associated with greater interference with daily activities due to dysmenorrhea. ∙ Women with severe dysmenorrhea had longer sleep latency and lower sleep efficiency than those with mild dysmenorrhea. ∙ Women with mild dysmenorrhea had better sleep quality than those with moderate or severe dysmenorrhea.	High
Yasir et al. [45]	Female medical students	356	18–25y 21.01 ± 1.54	Cross-sectional	Simple question asking whether having sleep disturbances as associated symptoms of dysmenorrhea	n/a	Simple question asking whether having dysmenorrhea	n/a	64% of women with dysmenorrhea were affected by sleep disturbance.	Low
Zeru et al. [21]	Female undergraduate students	620	18–26y 20.6 ± 1.4	Cross-sectional	Sleep duration (≤ 5 h; 6–7 h; ≥ 8 h)	n/a	n/a	Outside the regular menstrual cycle limit as follow: ∙ Frequency of menstruation: 24–38 days, ∙ Duration of bleeding ≤ 8 days ∙ Cycle to cycle variation over the last one year < 10 days ∙ The individual perception on the amount is normal	Compared to the students who slept ≥ 8 h, ≤ 5 sleep hours increased 5 times risk of having menstrual irregularity (AOR = 5.4, 95% CI [2.98–9.98]), and 6–7 sleep hours increased 1.9 times risk of having menstrual irregularity (AOR = 1.9. 95% CI [1.29 − 2.91]).	Moderate

*Note.* Abbreviations: SD = Standard deviation, PMS = Premenstrual syndrome, PMDD = Premenstrual dysphoric disorder, n/a = Not applicable, VAS = Visual analog scale, PSD = Pittsburgh Sleep Diary, DSR = Daily Symptom Rating, DSM-IV = Diagnostic and Statistical Manual of Mental Disorders, fourth edition, SWS = Slow wave sleep, ESS = Epworth Sleepiness Scale, ISI = Insomnia Severity Index, PSQI = Pittsburgh Sleep Quality Index, OR = Odds ratio, CI = Confidence interval, PMTS = Premenstrual Tension Observer Rating Scale, PSST = Premenstrual Symptoms Screen Tool, DRSP = Daily Record of Severity of Problems, PMSS = Premenstrual Syndrome Scale, S-PAF = Shortened Premenstrual Assessment Form, PSS = Premenstrual Syndrome Scale, DSM-IV-TR = Diagnostic and Statistical Manual of Mental Disorders, fourth edition, text revision, PIRS = Pittsburgh Insomnia Rating Scale, PMDDSQ = Premenstrual Dysphoric Disorder Severity Questionnaire, MDQ = Menstrual Distress Questionnaire, PSI = Post-Sleep Inventory, SSQ = Self-screening Quiz, ICSD-II = International Classification of Sleep Disorders, second edition, CSD = Consensus Sleep Diary, AOR = Adjusted odds ratio

**Table 2:** Association between menstrual disturbances and sleep using Buysse’s sleep health framework

Dimensions and its definition in sleep health framework	Menstrual disturbances associations with sleep + positive, - negative, 0 null		
	Premenstrual syndrome (including PMDD)	Dysmenorrhea	Abnormal menstrual cycle (short or long)/Heavy bleeding
**S**atisfaction			
The subjective assessment of “good” or “poor” sleep	Worse sleep quality + [13, 32, 34, 39, 40, 43, 48, 50, 51, 53, 55, 57, 58] Perceived sleep quality 0 [42]	Worse sleep quality + [31, 33, 37, 40, 44, 54]	Worse sleep quality and abnormal menstrual cycle + [10, 54] Worse sleep quality and heavy bleeding + [10]
EEG (SWS or delta activity) ※The amounts of SWS or delta is correlated with sleep quality [24]	Decreased delta activity during sleep + [35] Decreased delta activity and increased theta activity + [39] Increased SWS + [41] Decreased SWS + [39] Change in SWS or delta activity 0 [36]		
Alertness during waking hours			
The ability to maintain attentive wakefulness (i.e., daytime sleepiness)	Greater daytime sleepiness + [53] Perceived daytime sleepiness 0 [50]	Greater daytime sleepiness + [31]	Perceived daytime sleepiness and abnormal menstrual cycle/heavy bleeding 0 [10]
Timing of sleep			
The placement of sleep within the 24-hour day	None	None	None
Efficiency			
The ease of falling asleep and/or returning to sleep			
• Increased WASO/SL (lower SE)	Increased WASO + [34] SL/WASO 0 [50]	Decreased sleep efficiency + [37] Increased SL + [44] Increased WASO + [33]	
• The subjective assessment of insomnia symptoms (ISI; DIS, DMS, EMA)	EMA + [34] Greater insomnia severity + [50, 57] Higher incidence of insomnia, greater insomnia severity + [52] Perceived insomnia symptoms 0 [13]	Greater insomnia severity + [31, 33, 44]	Higher incidence of insomnia, greater insomnia severity and abnormal menstrual cycle + [11] Perceived insomnia symptoms and abnormal menstrual cycle/heavy bleeding 0 [10]
Duration			
Total number of hours of sleep obtained per 24 h	Shorter sleep duration + [46] Shorter sleep duration + [56] Sleep duration 0 [55]	Shorter sleep duration + [33] Sleep duration 0 [22]	Short sleep duration (< 6 vs. > 8) and abnormal menstrual cycle + [47] Short sleep duration (< 5 vs. > 8) and abnormal menstrual cycle + [49] Short sleep duration (< 5, 6–7 vs. > 8) and abnormal menstrual cycle + [21] Short sleep duration (< 6 vs. > 9) and abnormal menstrual cycle/heavy bleeding + [10]
Unspecified	Greater sleep problems + [59]	Greater sleep problem + [45] Greater sleep problem + [15]	
	Frequent insomnia +; Greater daytime sleepiness + among women with any type of menstrual disturbances (PMS and/or dysmenorrhea, and/or heavy bleeding) [38]		

*Note.* PMDD = Premenstrual dysphoric disorder; SWS = Slow wave sleep; WASO = Wake after sleep onset; SL = Sleep latency; SE = Sleep efficiency; ISI = Insomnia severity index; DIS = Difficulty initiating sleep; DMS = Difficulty maintaining sleep; EMA = Early morning awakenings

### Sleep health framework

Buysee’s sleep health framework consists of five dimensions of sleep (**SATED**: **S**atisfaction, **A**lertness during waking hours, **T**iming of sleep, **E**fficiency, and Sleep **D**uration) to understand specific dimensions of sleep and its association with specific health outcomes [24]. Five dimensions in Buysse’s sleep health framework were applied to comprehensively understand the association between specific dimensions of sleep and menstrual disturbances. **S**atisfaction is defined as: (1) the subjective assessment of good or poor sleep; and (2) amounts of slow wave sleep (SWS) or delta activity during sleep measured by polysomnography. The amounts of SWS or delta activity during sleep is correlated with sleep quality [24]. **A**lertness during waking hours is the ability to maintain attentive wakefulness. Daytime sleepiness reduces alertness during waking hours. **T**iming of sleep is where sleep is placed within the 24-hour day. **E**fficiency is the ease of falling asleep and/or returning to sleep. Increased wake after sleep onset or sleep latency is associated with lower sleep efficiency, and with the subjective assessment of insomnia symptoms including difficulty initiating sleep, difficulty maintaining sleep, or early morning awakenings, which are included in the dimension of efficiency. **D**uration is the total number of hours of sleep obtained per 24 h [24].

### Study quality assessment

The quality assessment of the included studies was performed using the Joanna Briggs Institute (JBI) Critical Appraisal Checklist for Analytical Cross-Sectional Studies [29]. The JBI tool was selected as an assessment tool because most eligible studies included in this review had a cross-sectional design. Only one study had prospective longitudinal design [11]. Two authors (JB and BJ) independently assessed the quality of the included studies. Any disagreements were resolved through group discussions. The JBI tool consists of eight items evaluating methodological concepts such as participant inclusion criteria, measurement validity and reliability, confounding factors, and appropriate statistical analysis. The items were answered with three possible response categories (yes, no or unclear). An overall score was calculated for each included study by summing the number of “yes” responses. The maximum score was 8. A score of 1–4 was considered as low quality, from 5 to 6 as moderate and 7–8 as high quality [30]. No studies were excluded based on methodological quality.

## Results

A total of 35 studies were identified evaluating the association between menstrual disturbances (PMS and/or dysmenorrhea and/or abnormal menstrual cycle/heavy bleeding during periods) and sleep. Among menstrual disturbances, PMS extensively includes premenstrual dysphoria, premenstrual related negative affect symptoms, and PMDD. The identified studies were conducted in the multiple countries, including the United States, Canada, the Republic of South Africa, Australia, Taiwan, Turkey, Pakistan, India, Singapore, Malaysia, Iran, Brazil, Korea, Saudi Arabia, Ethiopia, Egypt, Ireland, and Japan. The sample size in the studies ranges between 9 and 11,648. Although most studies presented the mean ages or range of ages in study participants [10, 13, 15, 21, 22, 31–56], two studies did not have any information of age [57, 58], and two studies only reported approximate age in study participants [11, 59].

Detailed characteristics of study participants and measures of sleep and menstrual disturbances (PMS, dysmenorrhea, abnormal menstrual cycle/heavy bleeding during periods) are described in Table 1. While most studies explain how to measure menstrual disturbances, one study did not explain how to measure abnormal menstrual cycle [54] and one study measured menstrual disturbances comprehensively (having PMS and/or dysmenorrhea and/or abnormal menstrual cycle/heavy bleeding) [38]. One study evaluated sleep for PMS and dysmenorrhea [40]; one study for PMS and abnormal menstrual cycle [41]; one study for abnormal menstrual cycle and heavy bleeding [10] nineteen studies for PMS only [13, 32, 34–36, 39, 42, 43, 46, 48, 50–53, 55–59]; seven studies for dysmenorrhea only [15, 22, 31, 33, 37, 44, 45]; four studies for abnormal menstrual cycle only [11, 21, 47, 49].

Table 2 provides a detailed description of the sleep disturbances classified by related sleep health dimensions included in this review. Sleep quality issues (Satisfaction) were reported in most of the studies (66%), while other sleep characteristics including daytime sleepiness (Alertness during waking hours), the ease of falling asleep and/or returning to sleep (Efficiency), insomnia symptoms (Efficiency), and sleep duration (Duration) were reported in approximately half of the studies (57%). While majority of studies measured sleep characteristics using self-reported validated questionnaires or simple questions asking sleep characteristics, six studies (17%) used polysomnography to examine sleep architecture.

### Quality appraisal

The quality appraisal scores of individual studies included in this review are presented in Table 1 and Additional file 2. Seventeen studies (49%) were assessed as high quality, 14 (40%) as moderate, and 4 (4%) as low quality using the JBI tool. Most studies had clearly identified study participants, reported the setting, and used appropriate measurement. However, 16 studies (46%) identified and dealt with confounding factors.

### PMS and sleep health

Table 2 summarized the association between PMS and sleep health dimensions. Twenty-one studies [13, 32, 34–36, 39–43, 46, 48, 50–53, 55–59] explained the association between PMS and sleep disturbances. PMS including premenstrual dysphoria, premenstrual related negative affect symptoms, and PMDD was associated with sleep disturbances in four dimensions of sleep health including **S**atisfaction, **A**lertness during waking hours, **E**fficiency, and **D**uration (Table 2).

Fifteen studies provided evidence with poor sleep quality (Satisfaction) and PMS. Worse sleep quality (Satisfaction) was associated with women who had PMS compared to those without PMS [34, 36, 39, 50, 51, 53]. Women who had PMS were more likely to report poor sleep quality than those without PMS [32, 43, 58], and having poor sleep quality increased the risk of having PMS [43, 53]. These findings were consistent with women who had premenstrual dysphoria [40] or premenstrual related negative affect symptoms [35], or PMDD [41, 46, 48] with the reporting having worse sleep quality than those without these symptoms. The severity of PMS also had positive association with poor sleep quality [55]. Worse sleep quality was found in those with moderate PMS compared to those with mild PMS or without PMS [13, 57]. As physiological markers, the amount of SWS or delta activity from polysomnography could be measures for good sleep quality (Satisfaction) because of their positive correlation with sleep quality [24]. Two studies found significant decrease of delta activity in women with PMS [39] and premenstrual related negative affect symptoms [35] compared to those without. However, increased SWS activity was found in women with PMDD [41]. While most studies reported that PMS was significantly associated with poor sleep quality, there were no significant differences in SWS or delta activity and the severity of self-reported sleep quality between women with severe PMS and those without PMS [36, 42].

Women with PMS experienced greater daytime sleepiness (Alertness during waking hours) than those without PMS [53]. In addition, women who had PMS tended to have longer times to get back to sleep after frequent awakenings (i.e., increased wake after sleep onset) during sleep, and experienced frequent early morning awakenings (Efficiency) compared to those without PMS [34]. Two studies compared women with moderate levels of PMS, mild levels of PMS, and without PMS [13, 57]. Although women with moderate levels of PMS suffered from greater insomnia (Efficiency) compared to other groups [13, 57], only one study had statistically significant results [57]. In one epidemiology study, women with PMS experienced greater insomnia symptoms (Efficiency) than those without PMS, although there were no differences in the level of daytime sleepiness (Alertness during waking hours), sleep latency, and wake after sleep onset (Efficiency) between women with PMS and without PMS [50]. In women with PMDD, the incidence of insomnia was higher than those without PMDD, and greater severity of PMDD was associated with greater insomnia [52]. In terms of sleep duration, women who had PMDD had significantly shorter sleep duration (Duration) than those without PMDD [46]. Less than 6 h at night was associated with worse PMS [56], but longer sleep duration was not significantly correlated with the severity of PMS symptoms [55]. No studies reported findings about the dimension of timing of sleep in women with PMS.

### Dysmenorrhea and sleep health

Nine studies [15, 22, 31, 33, 37, 40, 44, 45, 54] explained the association between dysmenorrhea and sleep disturbances. Among these studies, having dysmenorrhea was associated with worse sleep quality (Satisfaction) [31, 33, 37, 40, 44, 54], excessive daytime sleepiness (Alertness during waking hours) [31], lower sleep efficiency and greater insomnia symptoms (Efficiency) [31, 33, 37, 44], and shorter total hours of sleep (Duration) [22, 33] (Table 2). In three studies, women with dysmenorrhea tended to have worse sleep quality (Satisfaction) than those without dysmenorrhea [31, 37, 40]. Among women with dysmenorrhea, increased severity of dysmenorrhea was associated with worse sleep quality and feeling unrested after waking up (Satisfaction) [33, 44]. Among women of reproductive age, worse sleep quality (Satisfaction) during the coronavirus disease 2019 (COVID-19) pandemic was associated with an increased number of painful periods [54].

Those women who had dysmenorrhea experienced greater daytime sleepiness (Alertness during wakefulness) than those without dysmenorrhea [31]. Women with dysmenorrhea experienced greater insomnia symptoms [31, 44] and insomnia severity (Efficiency) was positively associated with the severity of dysmenorrhea [31, 33, 44]. Conversely, women with dysmenorrhea experienced worse insomnia symptoms than those without dysmenorrhea [33]. In addition, dysmenorrhea was associated with the increase in sleep latency and in time to get back to sleep after waking during sleep (Efficiency) [44], which in turn was associated with a decrease in sleep efficiency (Efficiency) [37, 44]. One study found a significant relationship between women with dysmenorrhea as having shorter sleep duration [33] when compared with those without dysmenorrhea; however, there was no significant relationship between sleep duration and the severity of dysmenorrhea among women with no history of a previous pregnancy and/or childbirth [22]. No studies reported findings about the dimension of timing of sleep in women with dysmenorrhea.

### Abnormal menstrual cycle/heavy bleeding and sleep health

Six studies [10, 11, 21, 47, 49, 54] reported relationship between abnormal menstrual cycle and sleep disturbances (Table 2). Among these studies, one study [10] additionally examined the association between heavy bleeding during period and sleep disturbances. Poor sleep quality (Satisfaction) increases the risk of abnormal menstrual cycle change (short, long or missed periods) [10, 54]. One longitudinal study found that having insomnia (Efficiency) increased the risk of menstrual cycle irregularity more than 2 times higher over a year in both women with abnormal menstrual cycle and without this symptom at baseline [11]. Compared to women who had regular sleep duration (more than 8 or 9 h), those with short sleep duration (less than 6 or 5 h; Duration) were more likely to have abnormal menstrual cycle [10, 21, 47, 49] and heavy bleeding during periods [10]. The severity of daytime sleepiness (Alertness during waking hours) and insomnia (Efficiency) were not associated with abnormal menstrual cycle and heavy bleeding during periods [10]. No studies reported findings about the dimension of timing of sleep.

### Unspecified studies which explained the association between sleep and menstrual disturbances

Table 2 summarized the results of studies, which were not able to specify the dimension of sleep health or type of menstrual disturbances. About half (57%) of women with PMS had sleep problems and the severity of PMS was associated with greater sleep problems [59]. Similarly, among women with dysmenorrhea, 43% [45] or 63% [15] experienced sleep disturbances as an associated symptom of dysmenorrhea. Women with any type of menstrual disturbances (i.e., PMS and/or bothersome cramping and/or heavy bleeding during periods) were more likely to report frequent insomnia or excessive daytime sleepiness than those without any symptoms during the past 12 months [38]. Regardless of sleep health dimensions, women with PMS had a lower saturation of peripheral oxygen recorded by polysomnography than controls, which indicates the high risk of obstructive sleep disorder [50]. Women with primary dysmenorrhea were more likely to be diagnosed with restless leg syndrome than controls [31].

## Discussion

This study provides a systematic review of studies that examine the association between sleep and menstrual disturbances (PMS, dysmenorrhea and/or abnormal menstrual cycle and heavy bleeding during periods). Comprehensively, most of the evidence suggested that having PMS, dysmenorrhea, abnormal menstrual cycle, and heavy bleeding during periods are associated with sleep disturbances following most dimensions of the sleep health framework. Women with menstrual disturbances had poor sleep quality (Satisfaction), had difficulty staying awake or alert during daytime (Alertness during waking hours), had longer sleep latency and waketime after sleep onset, or early morning awakenings, which refers to insomnia symptoms (Efficiency), and had short sleep duration (Duration). In addition, the severity of menstrual disturbances is positively associated with worse sleep quality (Satisfaction), greater daytime sleepiness (Alertness during waking hours) and insomnia symptoms (Efficiency), and/or shorter sleep duration (Duration). However, timing of sleep (e.g., the preference of time for go to sleep and wake up; chronotype) was not examined in women with menstrual disturbances. Overall, there is evidence to suggest that sleep disturbances may be linked to menstrual disturbances in women.

The potential mechanism between sleep and menstrual disturbances is unknown. There is a possibility that sleep disturbances may affect circadian rhythms, which in turn may influence irregular menstrual cycles [60] because sleep and menstruation are both essential rhythmic physiological activities for women [2, 5]. To be specific, sleep disturbances may impair the secretion of gonadal hormones, which results in irregular menstrual cycles. Gonadal hormones in females, such as estrogens and progesterone, are produced by the signal from hypothalamus-pituitary-adrenal axis. The hypothalamus-pituitary-adrenal axis play an important role to control the entire body’s hormonal regulation. A function of hypothalamus-pituitary-adrenal axis is normally controlled by normal sleep circadian rhythms to produce gonadal hormone [61]. However, persons with sleep disturbances usually do not have regular circadian rhythms, which may be associated with irregular synthesis and secretion of female gonadal hormones [62]. A recent study reported that as sleep duration increased, estradiol and luteal phase progesterone concentrations increased [63]. Sleep disorders inhibit gonadotropin-releasing hormone secretion from the pituitary gland, thereby resulting in reduced reproduction of gonadal hormones [64, 65]. Maintaining regular circadian rhythm could be responsible for consistent regulation of menstrual-related hormonal.

The main biological cause of primary dysmenorrhea is the activation of prostaglandins because it induces the muscle and blood vessels to contract around the uterus. In a randomized control trial, the level of prostaglandins was significantly increased following the spontaneous pain intensity among participants under the condition of sleep deprivation [66]. This may suggest that activation of the prostaglandin system in response to sleep disturbances increases dysmenorrhea in women. In addition, the elevated levels of prostaglandin are associated with heavy menstrual bleedings because it is known to inhibit platelet and coagulation factors of aggregation [67]. As a result, women who experience dysmenorrhea and heavy bleeding during their periods may be more likely to have sleep disturbances, which can be mediated by the elevated levels of prostaglandin.

Sleep timing refers to chronotype which indicates the individual’s preference for going to bed and waking up a certain time during a 24-hour period. The evidence regarding the relationship between sleep timing and menstrual disturbances was not found in this review. However, shift workers involuntarily need to be forced to change their sleep timing regardless of their innate circadian rhythm. The shifted sleep-wake cycle is not synchronized with the individualized chronotype [68, 69] and previous studies have shown that rotating shift workers were more likely to experience irregular menstrual cycles or dysmenorrhea [65, 69–72]. However, these previous studies only considered the experience of shift work schedule but did not examine the variability of misalignment between the innate chronotype and the actual sleep-wake cycle in shift workers. It would be further evaluated whether greater variability in sleep timing and the innated chronotype is associated with menstrual disturbances among shift workers.

Despite the close association between sleep disturbances and menstrual disturbances, addressing and treatment of sleep disturbances was not considered as a therapeutic target for menstrual disturbances. The current interventions to reduce menstrual disturbances mainly focus on lifestyle modifications (e.g., dietary modification, exercise, stress reduction, yoga, acupuncture) [73–78]. In a recent randomized controlled trial, as both PMS symptoms and sleep duration were improved by lifestyle modifications in women with irregular menstrual cycle [75]. Given our systematic review, menstrual disturbances in women may be responsive to treatment that are useful for sleep disturbances.

There are several limitations to our study. Most included studies had cross-sectional design except one study [11]; therefore, we cannot assume a causal relationship between sleep and menstrual disturbances. In terms of measures, although there are the diagnostic criteria for PMS, dysmenorrhea, mensural cycle irregularity and heavy bleeding during menstruation, the definition of these menstrual disturbances are varied across the included studies. The studies included in the systematic review measured different aspects of menstrual disturbances and sleep, which varied widely. The heterogeneity among the included studies precluded the conducting of a meta-analysis. Therefore, although only a systematic literature review was conducted in this study, it has the strength of comprehensively examining previous studies on the relationship between menstrual disturbances and sleep, and identifying the research gaps and potential solutions. Most included studies used the self-reported measures to assess sleep. The self-reported measures are practical and cost-effective for research, but objective measures for sleep, such as actigraphy can increase the validity of the study. In addition, there was little and inconsistent evidence whether disturbed polysomnographic sleep measures for sleep quality (i.e., delta wave and SWS) are associated with menstrual disturbances. Therefore, laboratory studies using polysomnography measures needs to be considered to assess the dimension of satisfaction in sleep health framework in the future studies.

## Conclusions

This review highlights the effect of sleep disturbances on menstrual disturbances, including PMS, dysmenorrhea, abnormal menstrual cycle and heavy bleeding during periods. According to the Buysse’s sleep health framework, poor sleep satisfaction and efficiency, sleepiness during waking hours, and shorter sleep duration are associated with menstrual disturbances. Since there is currently no research on the relationship between sleep timing and menstrual disturbances, it would be valuable to conduct additional studies to examine whether greater variability in sleep timing and the innate chronotype is associated with menstrual disturbances. The findings from this review suggest that researchers and healthcare providers should consider sleep disturbances as a modifiable factor to manage menstrual disturbances. Moreover, this study provides preliminary evidence to develop sleep-related interventions among women who suffer from menstrual disturbances. Future study should incorporate the five dimensions of sleep health framework to comprehensively understand the multiple levels of sleep disturbances in women who suffer from menstrual disturbances.

### Electronic supplementary material

Below is the link to the electronic supplementary material.

Additional file 1: Search strategies

Additional file 2: Quality assessment of included studies

## Data Availability

The result of this systematic review was extracted from the data gathered and analyzed based on the stated methods and materials. All the relevant data are within the paper.

## List of Abbreviations

COVID-19: Coronavirus disease 2019
JBI: Joanna Briggs Institute
PMDD: Premenstrual Dysphoric Disorder
PMS: Premenstrual Syndrome
PRISMA: Preferred Reporting Items for Systematic Reviews and Meta-Analysis
SATED: Satisfaction, Alertness during waking hours, Timing of sleep, Efficiency, and Sleep Duration
SWS: Slow Wave Sleep
