# Supplementary material for: Menstrual disturbances and its association with sleep disturbances: a systematic review
Source: BMC Womens Health. 2023 Sep 1;23:470. doi: 10.1186/s12905-023-02629-0 (PMC10474748; doi:10.1186/s12905-023-02629-0)
Supplement: Supplementary file 1 — Additional file 1: Search strategies [file 12905_2023_2629_MOESM1_ESM.docx]

Additional file 1. Search strategies

| **Databases** | **Queries** |
| --- | --- |
| PubMed | (Dyssomnias[MH] OR sleep[MH] OR "Sleep Disorder*"[TIAB] OR "sleep disturbance*"[TIAB] OR "sleep wake disorder*"[TIAB]) AND (Menstruation Disturbances[MAJR] OR "Menstruation Disturbance*"[TIAB] OR "Menstrual disorders"[TIAB] OR "Irregular Menstruation"[TIAB] OR "Irregular Mense*"[TIAB] OR "Menstrual Irregularit*"[TIAB]) |
| Embase | ('sleep disorder'/exp OR 'sleep'/exp OR ((sleep NEAR/3 (disturbance* OR disorder* OR ‘wake disorder*’)):ab,ti,kw OR dyssomnia*:ab,ti,kw)) AND ('menstruation disorder'/exp OR ((menstrual OR menstruation OR mense*) NEAR/3 (irregularit* OR disorder* OR disturbance* OR dysfunction*)):ab,ti,kw) |
| PsycINFO | (exp Sleep/ OR exp Sleep wake disorders/ OR (sleep disorder* OR sleep disturbance*).mp.) AND (exp Menstrual Disorders/ OR exp Premenstrual Syndrome/ OR (Menstruation Disturbance* OR Irregular Menstruation OR Irregular Mense* OR Menstrual Irregualrit*).mp.) |
| CINAHL | ((MH "Dyssomnias+") OR (MH "Sleep+") OR (sleep N3 (disturbance* OR disorder* OR 'wake disorder*')) OR dyssomnia*) AND ((MH "Menstruation Disorders+") OR ((menstrual OR menstruation OR mense*) N3 (irregularit* OR disorder* OR disturbance* OR dysfunction*))) |
