# Supplementary material for: Menstrual disturbances and its association with sleep disturbances: a systematic review
Source: BMC Womens Health. 2023 Sep 1;23:470. doi: 10.1186/s12905-023-02629-0 (PMC10474748; doi:10.1186/s12905-023-02629-0)
Supplement: Supplementary file 2 — Additional file 2: Quality assessment of included studies [file 12905_2023_2629_MOESM2_ESM.docx]

Additional file 2. Quality assessment of included studies

(a) Quality of individual studies


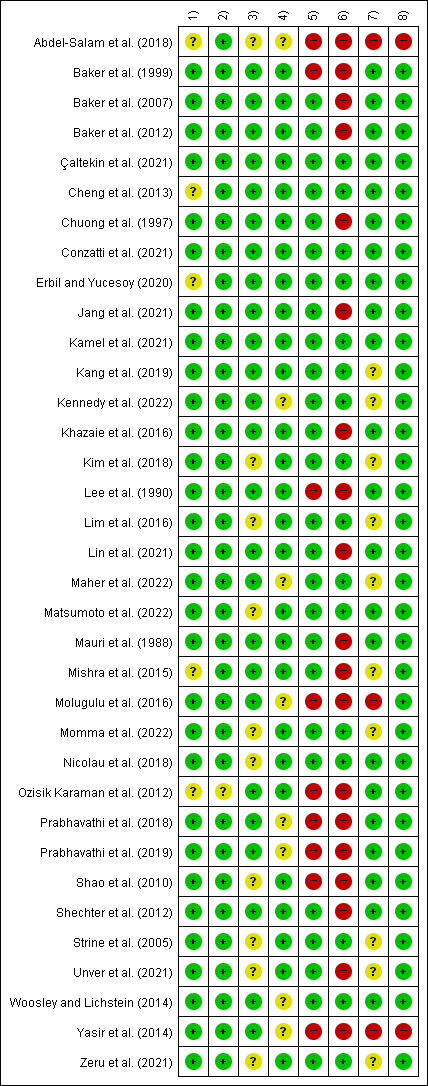


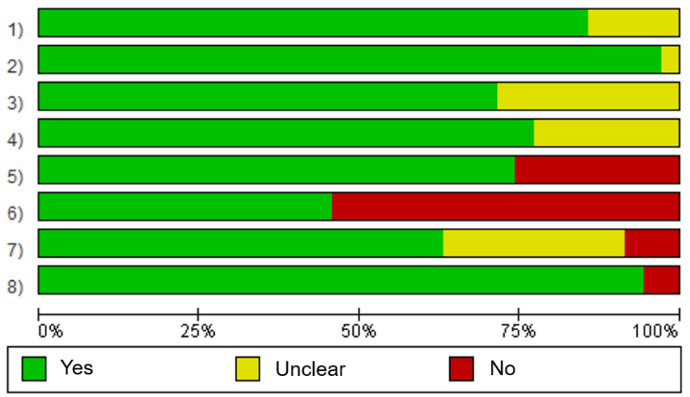
(b) Quality of overall studies

Note.

1) Were the criteria for inclusion in the sample clearly defined?

2) Were the study subjects and the setting described in detail?

3) Was the exposure measured in a valid and reliable way?

4) Were objective, standard criteria used for measurement of the condition?

5) Were confounding factors identified?

6) Were strategies to deal with confounding factors stated?

7) Were the outcomes measured in a valid and reliable way?

8) Was appropriate statistical analysis used?
